# Detection of Genuine and Posed Facial Expressions of Emotion: Databases and Methods

**DOI:** 10.3389/fpsyg.2020.580287

**Published:** 2021-01-15

**Authors:** Shan Jia, Shuo Wang, Chuanbo Hu, Paula J. Webster, Xin Li

**Affiliations:** ^1^State Key Laboratory of Information Engineering in Surveying Mapping and Remote Sensing, Wuhan University, Wuhan, China; ^2^Lane Department of Computer Science and Electrical Engineering, West Virginia University, Morgantown, WV, United States; ^3^Department of Chemical and Biomedical Engineering, West Virginia University, Morgantown, WV, United States

**Keywords:** facial expressions analysis, spontaneous expression, posed expression, expressions classification, countermeasure

## Abstract

Facial expressions of emotion play an important role in human social interactions. However, posed expressions of emotion are not always the same as genuine feelings. Recent research has found that facial expressions are increasingly used as a tool for understanding social interactions instead of personal emotions. Therefore, the credibility assessment of facial expressions, namely, the discrimination of genuine (spontaneous) expressions from posed (deliberate/volitional/deceptive) ones, is a crucial yet challenging task in facial expression understanding. With recent advances in computer vision and machine learning techniques, rapid progress has been made in recent years for automatic detection of genuine and posed facial expressions. This paper presents a general review of the relevant research, including several spontaneous vs. posed (SVP) facial expression databases and various computer vision based detection methods. In addition, a variety of factors that will influence the performance of SVP detection methods are discussed along with open issues and technical challenges in this nascent field.

## 1. Introduction

Facial expressions, one of the main channels for understanding and interpreting emotions among social interactions, have been studied extensively in the past decades (Zuckerman et al., 1976; Motley and Camden, 1988). Most existing research works have focused on automatic facial expression recognition based on Ekman's theories (Ekman and Keltner, 1997), which suggests six basic emotions universal in all cultures, including happiness, surprise, anger, sadness, fear, and disgust. However, are facial expressions always the mirror of our innermost emotions as we have believed for centuries? Recent research (Crivelli et al., 2015) has found that facial expressions do not always reflect our true feelings. Instead of reliable readouts of people's emotional states, facial expressions tend to be increasingly posed and even deliberately to show our intentions and social goals. Therefore, understanding the credibility of facial expressions in revealing emotions has become an important yet challenging task in human behavioral research especially among the studies of social interaction, communication, anthropology, personality, and child development (Bartlett et al., 1999).

In the early 2000s, Ekman's suggestion (Ekman, 2003) that a small number of facial muscles are not readily subject to volitional control, has laid the foundation for distinguishing between spontaneous and posed facial expressions. Ekman called these “reliable facial muscles” and claimed that activities of these muscles communicate the presence of specific emotions (Ekman, 2009). This set of muscles therefore became particularly trustworthy emotion-specific cues to identify genuine experienced emotions because they tend to be difficult to produce voluntarily. Early research of discriminating genuine facial expressions from posed ones heavily relied on a variety of observer-based systems (Mehu et al., 2012) targeting these muscles. Rapid advances in computer vision and pattern recognition especially deep learning techniques have recently opened up new opportunities for automatic and efficient identification of these cues for SVP facial expression detection. A variety of SVP facial expression detection methods (Valstar et al., 2006; Dibeklioglu et al., 2010; Wu et al., 2014; Huynh and Kim, 2017; Park et al., 2020), as well as publicly available databases (Wang et al., 2010; Pfister et al., 2011; Mavadati et al., 2016; Cheng et al., 2018), have been proposed for facial expression credibility analysis.

As of 2020, there has been no systematic survey yet to summarize the advances of SVP facial expression detection in the past two decades. To fill in this gap, we present a general review of this pioneering work as well as the most recent studies in this field including both existing SVP databases and automatic detection algorithms. Through literature surveys and analysis, we have organized existing SVP detection methods into four categories (action units, spatial patterns, visual features, and hybrid) and identified a number of factors that will influence the performance of SVP detection methods. Furthermore, we attempt to provide new insights into the remaining challenges and open issues to address in the future.

## 2. Spontaneous vs. Posed Facial Expression Databases

Early studies investigating facial expressions are mostly based on posed expressions due to the ease with which this data is collected where the subjects are asked to display or imitate each basic emotional expression. Spontaneous facial expressions, however, as natural expressions, need to be induced by various stimuli, such as odors (Simons et al., 2003), photos (Gajšek et al., 2009), and video clips (Pfister et al., 2011; Petridis et al., 2013). There have been several databases with single or multiple facial expressions collected to promote the research in automatic facial expression credibility detection. In this section, we first focus on databases with both spontaneous and posed facial expressions summarizing their details and characteristics. Then we review some databases with a single emotion category (either posed or spontaneous) but can provide rich data for detection of SVP facial expressions from different resources.

Table 1 provides an overview of existing SVP facial expression databases with both spontaneous and posed expressions. The MMI facial expression database (Pantic et al., 2005) was first collected with only posed expressions for facial expression recognition. Later, data with three spontaneous expressions (disgust, happiness, and surprise) were added with audio-visual recordings based on video clips as stimuli (Valstar and Pantic, 2010). USTC-NVIE (Wang et al., 2010) is a visible and infrared thermal SVP database. Six spontaneous emotions consisting of image sequences from onset to apex^1^ were also induced by screening carefully selected videos, while the posed emotions consist of apex images. CK+ database (Lucey et al., 2010), UvA-NEMO (Dibeklioğlu et al., 2012), and MAHNOB database (Petridis et al., 2013) all focused on the subject's smile, which is the easiest emotional facial expression to pose voluntarily. Specifically, the video sequences in the CK+ database were fully coded based on the Facial Action Coding System (FACS) (Ekman, 1997) with facial action units (AUs) as emotion labels, while videos in MAHNOB recorded both smiles and laughter with microphones, visible, and thermal cameras.

**Table 1:** Description of SVP facial expression databases with both spontaneous and posed facial expressions (AU-Action Units).

**Dataset**	**Expression**	**#Sub**	**#M/F**	**Age**	**#P/S**	**Format**	**Feature**	**References**
MMI	Multiple	25	13/12	20–32	2489/392	Video	Audio-visual; single and combinations of AUs	Valstar and Pantic, 2010
USTC-NVIE	Multiple	215	157/58	17–31	-/-	Frame	Visible + infrared thermal images	Wang et al., 2010
CK+	Smile	210	65/145	18–50	593/122	Frame	Multiple posed expressions, only un-posed smile, FACS coded	Lucey et al., 2010
SPOS Corpus	Multiple	7	4/3	/	51/147	Frame	Visible + infrared	Pfister et al., 2011
UvA-NEMO	Smile	400	215/185	8–76	643/597	Video	The largest smile database	Dibeklioğlu et al., 2012
MAHNOB	Smile	22	12/10	~28	563/101	Video	Audio-visual, thermal recording	Petridis et al., 2013
BioVid	Pain	90	45/45	18–65	630/8700	Video	Biopotential signals, depth information	Walter et al., 2013
DISFA	Multiple	27	15/12	18–50	0/54	Video	AU labels and landmarks	Mavadati et al., 2013
DISFA+	Multiple	9	4/5	18–50	644/0	Frame	AU labels, 42 facial actions	Mavadati et al., 2016
SASE-FE	Multiple	50	-/-	19–36	300/300	Video	3 subsets	Wan et al., 2017
4DFAB	Multiple	180	120/60	5–75	-/-	4D video	Dynamic high-resolution 3D faces, 79 face landmarks	Cheng et al., 2018

**Figure 1:**
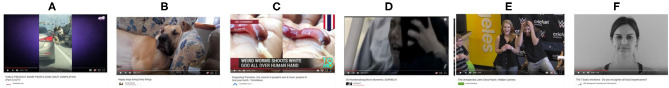
Screenshots of video clips to induce specific emotions in SASE-FE database (Copyright permission is obtained from Kulkarni et al., 2018). These video stimuli contain either specific scenes (such as **A,D,E**), objects (such as **B,C**), or the target emotions themselves (such as **D–F**) for emotion elicitation.

The SPOS Corpus database (Pfister et al., 2011) included six basic SVP emotions, with labels for onset, apex, offset and ends with two annotators according to subjects' self-reported emotions. The BioVid dataset (Walter et al., 2013) specifically targeted pain with heat stimulation, and both biosignals (such as skin conductance level [SCL], electrocardiogram [ECG], electromyogram (EMG), and electroencephalography [EEG]) and video signals were recorded. DISFA and DISFA+ databases (Mavadati et al., 2013, 2016) contain spontaneous and posed facial expressions, respectively, with 12 coded AUs labeled using FACS and 66 landmark points. In addition to basic facial expressions, DISFA+ also includes 30 facial actions by asking participants to imitate and pose specific expressions. Originally proposed for the ChaLearn LAP Real vs. Fake Expressed Emotion Challenge in 2017, the SASE-FE database (Wan et al., 2017; Kulkarni et al., 2018) collected six expressions by asking participants to pose artificial facial expressions or showing participants video clips to induce genuine expressions of emotion. Figure 1 illustrates several examples of video clips selected by psychologists to induce specific emotions in this database. Most recently, a large scale 4D database, 4DFAB (Cheng et al., 2018), was introduced with 6 basic SVP expressions, recorded in four different sessions spanning over a 5-year period. This is the first work to investigate the use of 4D spontaneous behaviors in biometric applications.

We further introduce some databases widely-used in the emotion detection field with either posed or spontaneous facial expressions, which can provide rich data with different resources for SVP facial expression detection. Table 2 shows the details of these popular facial expression databases. The Karolinska Directed Emotional Face (KDEF) dataset (Lundqvist et al., 1998) contains 4,900 images (562 × 762 pixels) from 70 subjects, each with seven posed emotional expressions taken from five different angles. Oulu-CASIA (Zhao et al., 2011) is a NIR-VIS posed expression database with 2,880 image sequences collected from 80 subjects. Six basic expressions are recorded in the frontal direction under three different lighting conditions. Another widely-used posed expression dataset is the Japanese Female Facial Expressions (JAFFE) (Lyons et al., 2020), which consists of 213 grayscale images with seven emotions from 10 Japanese females. In terms of spontaneous expressions, the MPI dataset (Kaulard et al., 2012) collects 55 expressions with high diversity in three repetitions, two intensities, and three recording angles from 19 German subjects. The Binghamton-Pittsburgh 3D Dynamic Spontaneous (BP4D-Spontaneous) (Zhang et al., 2014) dataset collects both 2D and 3D videos of 41 participants from different races.

**Table 2:** Description of facial expression databases with either spontaneous (S) or posed (P) facial expressions.

**Dataset**	**Expression**	**#Sub**	**#M/F**	**Age**	**#P/S**	**Format**	**Feature**	**References**
KDEF	Multiple	70	35/35	20–30	P-4900	Image	5 different angles	Lundqvist et al., 1998
Oulu-CASIA	Multiple	80	59/21	23–58	P-2880	Image	Visible + infrared	Zhao et al., 2011
JAFFE	Multiple	10	0/10	/	P-213	Image	Japanese female, grayscale images	Lyons et al., 2020
MPI	Multiple	19	9/10	20–30	S-1045	Image	German participants, high diversity	Kaulard et al., 2012
BP4D-Spontaneous	Multiple	41	18/23	18–29	S-328	Video	Multiple races, both 2D + 3D videos	Zhang et al., 2014

There are also facial expression databases with rich data collected in the wild, such as the Real-world Affective Database (RAF-DB) (Li S. et al., 2017), Real-world Affective Faces Multi Label (RAF-ML) (Li and Deng, 2019), and Aff-wild database (Kollias et al., 2019), or collected from movies, such as the Acted Facial Expressions in the Wild (AFEW) and its static subset Static Facial Expressions in the Wild (SFEW). These kinds of data are of great variability to reflect the real-world situations (please refer to recent surveys [Huang et al., 2019; Saxena et al., 2020] for more details about these facial expression databases).

## 3. Detection of Genuine and Posed Facial Expressions

Posed facial expressions, due to their deliberate and artificial nature, always differ from genuine ones remarkably in terms of intensity, configuration, and duration, which have been explored as distinct features for SVP facial expression recognition. Based on different distinct clues, we classify existing methods into four categories: *muscle movement (action units) based, spatial patterns based, texture features based, and hybrid methods*.

### 3.1. Muscle Movement (Action Units) Based

**Figure 2:**
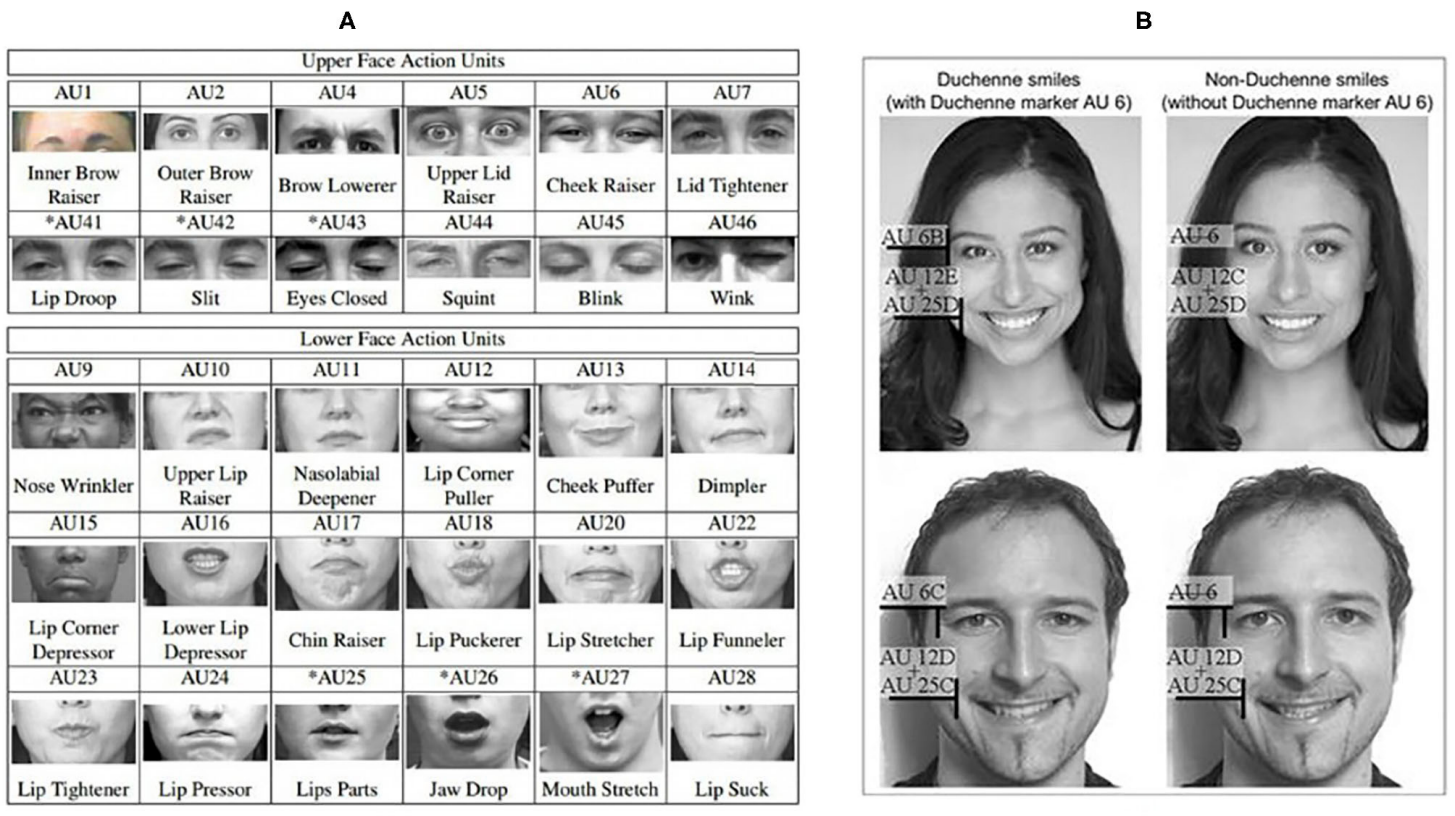
Examples of Facial Action Coding System (FACS) Action Units (AUs) **(A)** Upper and lower face AUs (Copyright permission is obtained from la Torre De et al., 2015), **(B)** Different AUs in Duchenne (genuine) smiles (AU 6, 12, 25) and non-Duchenne smiles (AU12, 25) (Copyright permission is obtained from Bogodistov and Dost, 2017).

Early research on distinguishing genuine facial expressions from posed ones rely a lot on the analysis of facial muscle movements. This class of methods is based on the assumption that some specific facial muscles are particularly trustworthy cues due to the intrinsic difficulty of producing them voluntarily (Ekman, 2003). In these studies, the Facial Action Coding System (FACS) (Ekman and Rosenberg, 2005) is the most widely-used tool for decomposing facial expressions into individual components of muscle movements, called Action Units (AUs), as shown in Figure 2A. Several studies have explored the differences of muscle movements (AUs) in spontaneous and posed facial expressions, including the AU's amplitude, maximum speed, and duration (please refer to Figure 2B for an example).

It is known that spontaneous smiles have a smaller amplitude, but a larger and more consistent relation between amplitude and duration than deliberate, posed smiles (Baloh et al., 1975). Based on this observation, a method in Cohn and Schmidt (2003) used timing and amplitude measures of smile onsets for detection and achieved the recognition rate of 93% with a linear discriminant analysis classifier (LDA). The method in Valstar et al. (2006) was the first attempt to automatically determine whether an observed facial action was displayed deliberately or spontaneously. They proposed to detect SVP brow actions based on automatic detection of three AUs (AU1, AU2, and AU4) and their temporal segments (onset, apex, offset) produced by movements of the eyebrows. Experiments on the combined databases have achieved 98.80% accuracy. Later works (Bartlett et al., 2006, 2008) extracted five statistic features (median, maximum, range, first-to-third quartile difference) of 20 AUs in each video segment for classification of posed and spontaneous pain. They reported a 72% classification accuracy on their own dataset. To detect SVP smiles, the method in Schmidt et al. (2009) quantified lip corner and eyebrow movement during periods of visible smiles and eyebrow raises, and found maximum speed and amplitude were greater and duration shorter in deliberate compared to spontaneous eyebrow raises. Aiming at multiple facial expressions, the method (Saxen et al., 2017) generated a 440-dimensional statistic feature space from the intensity series of seven facial AUs, and increased the performance to 73% by training an ensemble of Rank SVMs on the SASE-FE database. Alternatively, recent work in Racoviţeanu et al. (2019) used the AlexNet CNN architecture on 12 AU intensities to obtain the features in a transfer learning task. Training on the DISFA database, and testing on SPOS, the method achieved an average accuracy of 72.10%. A brief overview of these methods has been shown in Table 3.

**Table 3:** A brief overview of muscle movement based spontaneous vs. posed (SVP) detection methods (AU-action unit; LDA-linear discriminant analysis [classifier]; SVM-support vector machine).

**References**	**Method (features)**	**Expression**	**AU**	**Classification**	**Database**	**Accuracy (%)**
Cohn and Schmidt, 2003	Using timing and amplitude measures of smile onsets	Smile	6, 12, 15, 17	LDA	Self-collected	93.00
Valstar et al., 2006	Temporal dynamics of brow actions based on AUs and their temporal segments (onset, apex, offset)	Multiple (6)	1, 2, 4	Relevance Vector Machine	MMI+DS118+ CK+(262)	90.80
Bartlett et al., 2008	Statistic features of 20 AUs in each video segment	Pain	1, 2, 4–7, 9, 10, 12, 14, 15, 17, 18, 20, 23–26	Non-linear SVM	Self-collected	72.00
Schmidt et al., 2009	Maximum speed and amplitude of movement onset of lip corner and eyebrow; AFIA to measure movement	Smile	6, 12, 14, 15, 17, 23, 24, 50	(-)	Self-collected	(-)
Saxen et al., 2017	statistic features (440-dimensional) from the intensity time series of 7 facial AUs	Multiple (6)	1, 2, 4, 6, 9, 12, 25	Rank SVMs	SASE-FE	73.00
Racoviţeanu et al., 2019	AlexNet CNN architecture on 12 AU intensities to obtain the features in a transfer learning manner	Multiple (6)	1, 2, 4-6, 9, 12, 15, 17, 20, 25, 26	SVM	DISFA, SPOS	72.10

### 3.2. Spatial Patterns Based

This category of methods aim at exploring spatial patterns based on temporal dynamics of different modalities, such as facial landmarks and shapes of facial components. A multimodal system based on fusion of temporal attributes including tracked points of the face, head, and shoulder were proposed in Valstar et al. (2007) to discern posed from spontaneous smiles. Best results were obtained with late fusion of all modalities of 94% on 202 videos from the MMI database. Specifically regarding smiles, a study in Van Der Geld et al. (2008) analyzed differences in tooth display, lip-line height, and smile width between SVP smiles. They revealed several findings in SVP smiling differences. For example, maxillary lip-line heights in genuine smiles were significantly higher than those in posed smiles. When compared to genuine smiling, the tooth display in the (pre)molar area of posed smiling decreased by up to 30%, along with a significant reduction of smile width. Spatial patterns based on distance and angular features for eyelid movements were used in Dibeklioglu et al. (2010) and achieved 85 and 91% accuracy in discriminating SVP smiles on the BBC and CK databases, respectively. Based on fusing dynamics signals of eyelids, cheeks, and lip corners, more recent methods (Dibeklioğlu et al., 2012, 2015) achieved promising detection results on several SVP smile databases.

In multiple SVP facial expression detection studies, different schemes for spatial pattern modeling were used, including Restricted Boltzmann Machines (RBMs) based in Wang et al. (2015, 2016), Latent Regression Bayesian Network based in Gan et al. (2017), and interval temporal restricted Boltzmann machine (IT-RBM) in Wang et al. (2019). Results on several SVP databases confirmed the discriminative power and reliability of spatial patterns in distinguishing genuine and posed facial expressions. Similarly, Huynh and Kim (2017) used mirror neuron modeling and Long-short Term Memory (LSTM) (Hochreiter and Schmidhuber, 1997) with parametric bias to extract features in the spatial-temporal domain from extracted facial landmarks, and achieved 66% accuracy on the BABE-FE database. Table 4 includes an overview of these spatial pattern based detection methods.

**Table 4:** A brief overview of spatial patterns based spontaneous vs. posed (SVP) detection methods (SVM-support vector machine; RBM-Restricted Boltzmann Machines).

**References**	**Method (features)**	**Expression**	**Classification**	**Database**	**Accuracy (%)**
Valstar et al., 2007	Fusing temporal dynamics of head (6 features), face (12 points), and shoulder (5 points) modalities	Smile	GentleSVM-Sigmoid	MMI (202)	94.00
Van Der Geld et al., 2008	Analyzing tooth display, lip position and smile width in a dental perspective	Smile	(-)	Self-collected	(-)
Dibeklioglu et al., 2010	Distance-based and angular features for eyelid movements	Smile	Naive Bayes	BBC, CK	85.00, 91.00
Dibeklioğlu et al., 2012	Fusing the dynamics of eyelid, cheek, and lip corner movements	Smile	linear SVM	BBC, SPOS, UvA-NEMO	90.00, 75.00, 87.02
Dibeklioğlu et al., 2015	Dynamics of eyelid, cheek, and lip corner movements	Smile	SVM	BBC, SPOS, UvA-NEMO, MMI	90.00, 78.75, 92.10, 89.69
Wang et al., 2015, 2016	Spatial pattern modeling based on multiple RBMs and incorporating gender and expression categories as privileged information	Multiple (6)	RBMs	SPOS, USTC-NVIE, MMI	76.07, 92.61, 89.79
Gan et al., 2017	Spatial patterns based on Latent Regression Bayesian Network from he displacements of facial feature points	Multiple (6)	Bayesian Networks	SPOS,USTC-NVIE	76.07, 98.74
Huynh and Kim, 2017	Spatial-temporal features using mirror neuron modeling and LSTM with parametric bias from facial landmarks	Multiple (6)	Gradient boosting	SASE-FE	66.70
Wang et al., 2019	Universal spatial patterns and complicated temporalpatterns using IT-RBM dynamic model	Multiple (6)	Bayesiannetwork	SPOS, DISFA+	83.76, 96.24

### 3.3. Texture Features Based

Texture features based, such as Littlewort et al. (2009) designed a two-stage system to distinguish faked pain from real pain. It consisted of a detection stage for 20 facial actions using Gabor features and a SVM classification stage. The two-stage system achieved 88% accuracy on the UvA-NEMO dataset. Another method (Pfister et al., 2011) proposed a new feature (Completed local binary patterns from Three Orthogonal Planes [CLBP-TOP]), and fused the NIR and VIS modalities with the Multiple Kernel Learning (MKL) classifier, which achieved outstanding detection performance of 80.0% on the SPOS database. Finally, the approach in Gan et al. (2015) proposed to use pixel-wise differences between onset and apex face images as input features of a two-layer deep Boltzmann machine to distinguish SVP expressions. They achieved 84.62 and 91.73% on the SPOS and USTC-NVIE databases, respectively.

More recently, Mandal et al. (2016) explored several features, including deep CNN features, local phase quantization (LPQ), dense optical flow and histogram of gradient (HOG), to classify SVP smiles. With Eulerian Video Magnification (EVM) for micro-expression smile amplification, the HOG features outperformed other features with an accuracy of 78.14% on the UvA-NEMO Smile Database. Instead of using pixel-level differences, the method (Xu et al., 2017) designed a new layer named “comparison layer” for the deep CNN to generate high-level representations of the differences of onset and apex images, and verified its effectiveness on SPOS (83.34%) and USTC-NVIE (97.98%) databases. The latest work (Tavakolian et al., 2019) presents a Residual Generative Adversarial Network (R-GAN) based method to discriminate SVP pain expression by magnifying the subtle changes in faces. Experimental results have shown the state-of-the-art performance on three databases, with 91.34% on UNBC-McMaster (Lucey et al., 2011) with spontaneous pain expressions only, 85.05% on BiodVid, and 96.52% on STOIC (Roy et al., 2007) with posed expressions only. A brief overview of these methods is shown in Table 5.

**Table 5:** A brief overview of texture features based spontaneous vs. posed (SVP) detection methods.

**References**	**Method (features)**	**Expression**	**Classification**	**Database**	**Accuracy (%)**
Littlewort et al., 2009	Gabor features based	Pain	Gaussian SVM	UvA-NEMO	88.00
Pfister et al., 2011	Spatiotemporal local texture descriptor (CLBP-TOP), fusing the NIR and VIS modalities	Multiple (6)	MKL	SPOS	80.00
Liu and Wang, 2012	Temperature features from Infrared thermal images	Multiple (6)	Bayesian Networks	USTC-NIVE	76.70
Gan et al., 2015	A two-layer deep Boltzmann machine model based	Multiple (6)	Haarcascades	SPOS,USTC-NVIE	84.62, 91.73
Mandal et al., 2016	Several features: using CNN face features, LPQ, dense optical flow and HOG, and HOG with the best result	Smile	Linear SVM	UvA-NEMO	78.14
Xu et al., 2017	Learned features based on CNN from the difference of structural changes between the onset and apex images	Multiple (6)	Linear SVM	SPOS,USTC-NVIE	83.34, 97.98
Tavakolian et al., 2019	Encoding the dynamic and appearance of a video into an image map based on spatiotemporal pooling, then using R-GAN model for discrimination	Pain	Softmax	BioVid Heat Pain, STOIC, UNBC-McMaster	85.05, 96.52, 91.34

### 3.4. Hybrid Methods

Hybrid methods combined different classes of features for discriminating SVP facial expressions. Experiments on still images were conducted in Zhang et al. (2011) to show that appearance features (e.g., Scale-Invariant Feature Transform [SIFT] (Lowe, 2004)) play a significantly more important role than geometric features (e.g., facial animation parameters [FAP] (Aleksic and Katsaggelos, 2006)) on SVP emotion discrimination, and fusion of them leads to marginal improvement over SIFT appearance features. The average classification accuracy of six emotions is 79.4% (the emotion of surprise achieved the best result of 83.4% while anger had the worst at 77.2% accuracy) on the USTC-NVIE database. Sequential geometric features based on facial landmarks and texture features using HOG were combined in Li L. et al. (2017). A temporal attention gated model is designed for HOG features, combining with LSTM autoencoder (eLSTM) to capture discriminative features from facial landmark sequences. The proposed model performed well on most emotions on SASE-FE database, with an average accuracy of 68%. Mandal and Ouarti (2017) fused subtle (micro) changes by tracking a series of facial fiducial markers with local and global motion based on dense optical flow, and achieved 74.68% accuracy using combined features from the eyes and lips, slightly better than using only the lips (73.44%) and using only the eyes (71.14%) on the UvA-NEMO smile database. A different hybrid method in Kulkarni et al. (2018) combined learned static CNN representations from still images with facial landmark trajectories, and achieved promising performance not only in emotion recognition, but also in detecting genuine and posed facial expressions on the BABE-FE database with data augmentation (70.2% accuracy). Most recently, Saito et al. (2020) combined hardware (16 sensors embedded with the smart eye-wear) with a software-based method to extract geometric and temporal features to classify smiles into either “spontaneous” or “posed,” with an accuracy of 94.6% on their own database. See Table 6 for a brief summary of these hybrid SVP facial expression detection methods.

**Table 6:** A brief overview of hybrid methods for SVP detection.

**References**	**Method (features)**	**Expression**	**Classification**	**Database**	**Accuracy (%)**
Zhang et al., 2011	SIFT appearance based features and FAP geometric features	Multiple (6)	RBF SVM	USTC-NVIE	79.40
Li L. et al., 2017	Combining sequential geometric features based on facial landmarks and texture features using HOG	Multiple (6)	Sigmoid	SASE-FE	68
Mandal and Ouarti, 2017	Fusing subtle (micro) changes by tracking a series of facial fiducial markers with local and global motion based on dense optical flow	Smile	SVM	UvA-NEMO	74.68
Kulkarni et al., 2018	Combining learned static CNN representations from still images with facial landmark trajectories	Multiple (6)	Linear SVM	SASE-FE	70.20
Saito et al., 2020	Combining hardware (16 sensors embedded with the smart eyewear) with software-based method to get geometric and temporal features	Smile	Linear SVM	Self-collected	94.60

## 4. Discussions

The studies reviewed in the previous section indicate two key factors in the research on automatic SVP facial expression detection: *collection of SVP facial expression data* and *design of automatic detection methods*. We first discuss our findings in existing studies from the perspective of data collection and detection methodology, respectively. Then, we attempt to address several new challenging issues, including the necessity of collecting diverse datasets as well as performing a unified evaluation in terms of detection accuracy and generalizability.

### 4.1. Data Collection

The databases for SVP facial expressions play a significant role in benchmarking the effectiveness and practicality of different detection schemes. From Tables 2–5, it can be observed that the detection performance of the same detection method can vary widely in different databases. Such performance differences can be attributed to several uncertainty factors of data collection. As the collection process is mostly based on recording subjects' facial expressions when they are shown various stimuli (such as movie clips), the data size, subject selection, recording environment, and stimuli materials, all have a direct effect on the visual quality of collected video data. A detailed discussion of these influencing factors is included below:

- Several methods in Tables 3, 4 have illustrated worse detection performance on the smaller SPOS dataset (with seven subjects) than that on the larger USTC-NVIE dataset (with 215 subjects). This is because using a limited number of samples will not only limit the detection ability of data-driven based methods but also weaken the detection performance in practical applications.
- In terms of subjects, both age and gender will affect the SVP facial expression detection. Dibeklioğlu et al. (2012) has explored the effect of subject age by splitting the UvA-NEMO smile database into young (age <18) and adults (age ≥ 18 years), and found that eyelid-and-cheek features provided more reliable classification for adults, while lip-corner features performed better on young people. They further explored the gender effect in their completely automatic SVP detection method using dynamic features in different face regions and temporal phases (Dibeklioğlu et al., 2015). Experimental results showed that the correct classification rates on males were better than females for different facial region features. Such performance differences can be attributed to the fact that male subjects have more discriminative geometric features (distances between different landmark pairs) than female subjects. They also improved their detection performance by using age or gender as labels. Similarly, Wang et al. (2019) considered the influence of gender, and incorporated it as the input for performance improvement of their expression analysis model.
- The recording environment can vary greatly between studies in terms of the recording devices and lighting conditions. Most existing databases record images/videos of subjects in indoor controlled environments, which may limit the diversity of the data. In addition to visible images/videos, some studies have shown the impact of different modalities on improving the detection performance. Pfister et al. (2011) illustrated that the performance of fusion of NIR with visible images (80.0% accuracy) is better than using single NIR (78.2% accuracy) or visible images (72.0% accuracy) on the SPOS dataset. Although special devices are needed for data acquisition, the advantages of different modalities in revealing subtle features deserve further investigation. It is also plausible to combine the information contained in multiple modalities for further performance improvement.
- In the collection of spontaneous facial expressions, different stimuli are often selected by those generating the face databases or by psychologists to induce specific emotions from participants. The stimuli determine the categories of facial expressions included in databases directly, which will further influence the evaluation of the database. Due to the differences in activation of muscles, such as with different intensities and in different facial regions, each emotion has varying difficulty levels in SVP expression detection. For example, happiness and anger can activate obvious muscles around the eye and mouth regions, which has been widely studied for feature extraction. Based on appearance and geometric features, Zhang et al. (2011) found that surprise was the easiest emotion for their model to classify correctly (83.4% accuracy on USTC-NVIE), followed by happiness with 80.5% accuracy, while disgust was the most difficult (76.1% accuracy). Similarly, Kulkarni et al. (2018) achieved better results in detecting SVP happiness (71.05% accuracy) and anger (69.40% accuracy), but worse results for disgust (63.05% accuracy) and contempt (60.85% accuracy) on the SASE-FE dataset. On the contrary, Li L. et al. (2017) obtained the highest accuracy (80%) for both disgust and happy, while 50% for contempt on the SASE-FE dataset. Overall, SVP happiness is relatively easy to recognize.

### 4.2. Detection Methodology

Performance differences can also be observed on the same dataset among approaches in different categories. Generally speaking, the methodology for SVP facial expression detection involves several modules, including data pre-processing, features extraction, and classification. These modules are discussed separately below:

- As each emotion has its own discriminative facial regions, data pre-processing to extract specific facial regions is needed not only in emotion recognition but also in posed vs. genuine classification. The study in Zhang et al. (2011) has found that in SVP emotion detection, the mouth region is more important for sadness; the nose is more important for surprise; both the nose and mouth regions are important for disgust, fear, and happiness, while the eyebrows, eyes, nose, and mouth are all important for anger. Another study (Liu and Wang, 2012) also explored different facial regions, including the forehead, eyes, nose, cheek, and mouth. Experimental results have shown that the forehead and cheek performed better than the other regions for most facial expressions (disgust, fear, sadness, and surprise), while the mouth region performed the worst for most facial expressions. Moreover, fusing all these regions achieved the best performance. In SVP smile detection, it was observed in Dibeklioğlu et al. (2012) that the discriminative power of the eyelid region is better than the cheek and lip corners. A different study in Mandal and Ouarti (2017) has found that lip-region features (73.44% accuracy on UvA-NEMO) outperformed the eye-region features (71.14% accuracy), while the combined features performed the best with 74.68% accuracy for SVP smile detection. Overall, fusion of multiple facial regions can improve the detection performance over individual features. Besides, varying video temporal segments (i.e., onset, apex, and offset) for feature extraction also leads to different levels of performance. Several studies (Cohn and Schmidt, 2003; Dibeklioğlu et al., 2012) have demonstrated that the onset phase performs best among individual phases in SVP facial expression detection.
- It is clear that the features extracted for distinguishing between posed and spontaneous facial expressions play a key role in detection performance. Most methods have explored temporal dynamics of different features for effective detection. We can observe from Tables 2–5 that the detection performance varies greatly among different algorithms using the same database. The learned texture features from comparing the differences between images taken throughout the process of forming a facial expression proposed by Gan et al. (2015) and Xu et al. (2017) in Table 5 performed better than muscle movement and spatial pattern based methods on the SPOS database, while on the USTC-NIVE database and smile SVP database UvA-NEMO, spatial patterns based methods achieve slightly higher accuracy than texture features, and significantly higher than other kinds of methods. Overall, texture features based and spatial patterns based methods show more promising detection abilities; but there still lacks a consensus about which type of features will be optimal for the task of SVP detection.
- The classifier used also has a great effect on most classification tasks, which has also been explored by researchers in distinction between spontaneous and posed facial expressions. Dibeklioglu et al. (2010) assessed the reliability of their features with continuous HMM, k-Nearest-Neighbor (kNN), and the Naive Bayes classifier, and found that the highest classification rate was achieved by the Naive Bayes classifier on two datasets. Pfister et al. (2011) compared support vector machine (SVM), Multiple Kernel Learning (MKL), and Random Forest decision tree (RF) classifier, and found RF outperformed SVM and MKL based on CLBP-TOP features on the SPOS database. Dibeklioğlu et al. (2015) compared Linear Discriminant, Logistic Regression, kNN, Naive Bayes, and SVM classifiers on UvA-NEMO smile dataset, and showed the outstanding performance of the SVM classifier under all testing scenarios. Racoviţeanu et al. (2019) also used SVM, combined with a Hard Negative Mining (HNM) paradigm, to produce the best performance among RF, SVM, and Multi-Layer Perceptron (MLP) classifiers. Overall, as the most widely-used classifier, SVM can provide outstanding performance on several databases. Whether recently developed deep learning-based classifiers can achieve further performance improvement remains to be explored.

### 4.3. Challenges and Opportunities

Based on the summary of existing studies in SVP facial expression detection, we further discuss the challenges in both data collection and detection methods for developing an automatic SVP facial expression recognizer.

The database creation procedure in existing SVP facial expression databases is diverse and there is a lack of a unified protocol or guidelines for high quality database collection. Several general steps are involved in the process of data collection, including subject selection, stimulus selection, recording process, and data annotation. In addition to the influencing factors that have been studied in existing detection methods, (e.g., the data size, age and gender of subjects, recording environment and devices, and stimuli materials [please see the details in section 4.1]), there are more factors that may influence the database quality and deserve further investigation. For example, most databases ignore the external factors, such as personality or mood of the participants in subject selection. Some databases gave an introduction to the experimental procedure for the subjects in advance (e.g., the USTC-NVIE dataset), while some gave no instructions to subjects on how they should react and what the aim of the study was (such as the MAHNOB dataset). In terms of the stimulus selection, there is no detailed description on how the video clip stimuli were selected by collectors or psychologists. Besides the recording environment, the recording distance, shooting angle, and more importantly, the order setup for recording different emotions (e.g., to reduce the interaction of different emotions, neutral clips were shown to subjects between segments in USTC-NVIE), will all have an effect on the quality of collected data. Further, unlike posed emotions which subjects are asked to display, spontaneous emotions induced by specific video clips, are more difficult to label. In the DISFA and MMI datasets, the data were annotated based on FACS coding of facial muscle actions. The USTC-NVIE and SPOS Corpus databases used self-reported data of subjects as the real emotion labels. We believe that designing a protocol to unify these procedures to conduct deeper investigations to determine their influence on SVP emotion detection will contribute to higher-quality and more credible SVP expressions collection.

Collection of SVP facial expression datasets that are large-scale and diverse in subjects selection, emotion categories, and recording environment (such as fully unconstrained environments) are also in high demand to reflect the real-world situations. Existing databases with both spontaneous and posed facial expressions of the same subjects are limited in data size due to the difficulty of data collection. Moreover, the arbitrary movement of subjects, low resolution or occlusion (e.g., the person may not be looking directly at the camera) may occur in a realistic interaction environment, which has not been taken into consideration by existing databases. Taking advantage of rich datasets proposed for emotion detection is one alternative to help realize the full potential of data-driven detection methods. In addition, using the strength of the detection methods to aid the database creation is also worth exploring. For example, based on the findings that the data properties, such as subject age and gender can contribute to improvement of detection performance, the subject distribution in terms of age, gender, race, and even personality, should be considered in data collection, which will not only improve the data diversity, but also inspire researchers to design more effective and practical detection methods.

Another challenge is the lack of a unified evaluation standard (such as experimental data and annotation) for SVP facial expression detection. Therefore, it is difficult to compare the diverse methods reviewed in this paper on a common experimental setting. Although several studies have reported promising detection accuracy on specific datasets, they have observed the apparent gap of performance between posed facial expressions detection and genuine ones. For example, based on texture features, Liu and Wang (2012) found that it is much easier to distinguish all posed expressions (90.8% accuracy) than genuine ones (62.6%) using the USTC-NIVE database. Similarly, Mandal et al. (2016) also achieved higher classification accuracy of posed smiles than spontaneous ones (with over 10% gaps) on the UvA-NEMO dataset. However, two hybrid methods (Mandal and Ouarti, 2017; Kulkarni et al., 2018) both obtained higher accuracy in detecting genuine facial expressions than posed ones, with a 6% gap in methods (Mandal and Ouarti, 2017) on the UvA-NEMO Smile database, while an average of 7.9% gap in methods (Kulkarni et al., 2018) on the SASE-FE database. Such inconsistent differences, influenced by both feature extraction methods and databases, deserve to be reconciled in future research.

Furthermore, how to improve the generalization ability of SVP detection on multiple universal facial expressions, or improve the performance on a specific emotion based on its unique facial features, also deserves further investigation. Existing research can achieve promising detection performance on specific datasets under intra-dataset testing scenarios. However, few studies conduct cross-dataset testing evaluation (Cao et al., 2010) to show the detection robustness on facial expressions from unknown resources. Hybrid methods with fused features from multiple descriptors, multiple face regions, multiple image modalities, or multiple visual cues (such as including head movement and body gesture) require further investigation for the improvement of facial expression detection performance.

## 5. Conclusions

With the emerging and increasingly supported theory that facial expressions do not always reflect our genuine feelings, automatic detection of spontaneous and posed facial expressions have become increasingly important in human behavior analysis. This article has summarized recent advances of SVP facial expression detection over the past two decades. A total of sixteen databases and nearly thirty detection methods have been reviewed and analyzed here. Particularly, we have provided detailed discussions on existing SVP facial expression detection studies from the perspectives of both data collection and detection methodology. Several challenging issues have also been identified to gain a deeper understanding of this emerging field. This review is expected to serve as a good starting point for researchers who consider developing automatic and effective models for genuine and posed facial expression recognition.

One area that has not been covered by this paper is the 3D dynamic facial expression databases (Sandbach et al., 2012; Zhang et al., 2013). As 3D scanning technology (e.g., Kinect and LIDAR) rapidly advances, SVP detection from 3D, instead of 2D data, might become feasible in the near future. Can 3D information facilitate the challenging task of SVP facial expression detection? It remains to be explored. Research on SVP detection also has connections with other potential applications, such as Parkinson's disease (Smith et al., 1996), deception detection (Granhag and Strömwall, 2004), and alexithymia (McDonald and Prkachin, 1990). More sophisticated computational tools, such as deep learning based methods might help boost the research progress in SVP detection. It is likely that the field of facial expression recognition and affective computing will continue to grow in the new decade.

## Author Contributions

SJ and CH did the literature survey together. SJ and XL jointly worked on the write-up. PW and SW handled the revision. All authors contributed to the article and approved the submitted version.

## Conflict of Interest

The authors declare that the research was conducted in the absence of any commercial or financial relationships that could be construed as a potential conflict of interest.

## Footnotes

^1^Onset, apex, along with offset, and neutral, are four possible temporal segments of facial actions during the expression development (generally in the order of neutral→ onset→ apex→ offset → neutral). In the onset phase, muscles are contracting and changes in appearance are growing stronger. In the apex phase, the facial action is at a peak with no more changes in appearance. The offset phase describes that the muscles of the facial action are relaxing and the face returns to its original and neutral appearance, where there are no signs of activation of the investigated facial action.

## References

[B1] Aleksic P. S. Katsaggelos A. K. (2006). Automatic facial expression recognition using facial animation parameters and multistream hmms. IEEE Trans. Inform. Forens. Sec. 1, 3–11. 10.1109/TIFS.2005.863510

[B2] Baloh R. W. Sills A. W. Kumley W. E. Honrubia V. (1975). Quantitative measurement of saccade amplitude, duration, and velocity. Neurology 25, 1065–1065. 10.1212/WNL.25.11.1065 1237825

[B3] Bartlett M. Littlewort G. Vural E. Lee K. Cetin M. Ercil A. (2008). “Data mining spontaneous facial behavior with automatic expression coding,” in Verbal and Nonverbal Features of Human-Human and Human-Machine Interaction (Berlin; Heidelberg: Springer), 1–20. 10.1007/978-3-540-70872-8_1

[B4] Bartlett M. S. Hager J. C. Ekman P. Sejnowski T. J. (1999). Measuring facial expressions by computer image analysis. Psychophysiology 36, 253–263. 10.1017/S0048577299971664 10194972

[B5] Bartlett M. S. Littlewort G. Frank M. G. Lainscsek C. Fasel I. R. Movellan J. R. (2006). Automatic recognition of facial actions in spontaneous expressions. J. Multimed. 1, 22–35. 10.4304/jmm.1.6.22-35

[B6] Bogodistov Y. Dost F. (2017). Proximity begins with a smile, but which one? Associating non-duchenne smiles with higher psychological distance. Front. Psychol. 8:1374. 10.3389/fpsyg.2017.01374 28848483 PMC5554339

[B7] Cao L. Liu Z. Huang T. S. (2010). “Cross-dataset action detection,” in IEEE Computer Society Conference on Computer Vision and Pattern Recognition (San Francisco, CA), 1998–2005. 10.1109/CVPR.2010.5539875

[B8] Cheng S. Kotsia I. Pantic M. Zafeiriou S. (2018). “4DFAB: a large scale 4D database for facial expression analysis and biometric applications,” in Proceedings of the IEEE Conference on Computer Vision and Pattern Recognition (Salt Lake City), 5117–5126. 10.1109/CVPR.2018.00537

[B9] Cohn J. F. Schmidt K. L. (2003). “The timing of facial motion in posed and spontaneous smiles,” in International Journal of Wavelets Multiresolution & Information Processing 2.02, 121–132.

[B10] Crivelli C. Carrera P. Fernández-Dols J.-M. (2015). Are smiles a sign of happiness? Spontaneous expressions of judo winners. Evol. Hum. Behav. 36, 52–58. 10.1016/j.evolhumbehav.2014.08.009

[B11] Dibeklioğlu H. Salah A. A. Gevers T. (2012). “Are you really smiling at me? Spontaneous versus posed enjoyment smiles,” in European Conference on Computer Vision (Berlin; Heidelberg: Springer), 525–538. 10.1007/978-3-642-33712-3_38

[B12] Dibeklioğlu H. Salah A. A. Gevers T. (2015). Recognition of genuine smiles. IEEE Trans. Multimed. 17, 279–294. 10.1109/TMM.2015.2394777

[B13] Dibeklioglu H. Valenti R. Salah A. A. Gevers T. (2010). “Eyes do not lie: spontaneous versus posed smiles,” in Proceedings of the 18th ACM International Conference on Multimedia (Firenze), 703–706. 10.1145/1873951.1874056

[B14] Ekman P. (2003). Darwin, deception, and facial expression. Ann. N. Y. Acad. Sci. 1000, 205–221. 10.1196/annals.1280.010 14766633

[B15] Ekman P. (2009). Telling Lies: Clues to Deceit in the Marketplace, Politics, and Marriage (Revised Edition). New York, NY: WW Norton & Company.

[B16] Ekman P. Keltner D. (1997). “Universal facial expressions of emotion,” in Nonverbal Communication: Where Nature Meets Culture, eds U. Segerstrale and P. Molnar (Francisco, CA: California Mental Health Research Digest), 27–46.

[B17] Ekman P. Rosenberg E. (2005). What the Face Reveals: Basic and Applied Studies of Spontaneous Expression Using the Facial Action Encoding System (FACS). California, CA: Oxford University Press.

[B18] Ekman R. (1997). What the Face Reveals: Basic and Applied Studies of Spontaneous Expression Using the Facial Action Coding System (FACS). New York, NY: Oxford University Press.

[B19] Gajšek R. Štruc V. Mihelič F. Podlesek A. Komidar L. Sočan G. (2009). Multi-modal emotional database: AvID. Informatica 33, 101–106.

[B20] Gan Q. Nie S. Wang S. Ji Q. (2017). “Differentiating between posed and spontaneous expressions with latent regression bayesian network,” in Thirty-First AAAI Conference on Artificial Intelligence. San Francisco, CA.

[B21] Gan Q. Wu C. Wang S. Ji Q. (2015). “Posed and spontaneous facial expression differentiation using deep Boltzmann machines,” in 2015 International Conference on Affective Computing and Intelligent Interaction (ACII) (Xian: IEEE), 643–648. 10.1109/ACII.2015.7344637

[B22] Granhag P. A. Strömwall L. A. (2004). The Detection of Deception in Forensic Contexts. Cambridge University Press.

[B23] Hochreiter S. Schmidhuber J. (1997). Long short-term memory. Neural Comput. 9, 1735–1780. 10.1162/neco.1997.9.8.1735 9377276

[B24] Huang Y. Chen F. Lv S. Wang X. (2019). Facial expression recognition: a survey. Symmetry 11:1189 10.3390/sym11101189

[B25] Huynh X.-P. Kim Y.-G. (2017). “Discrimination between genuine versus fake emotion using long-short term memory with parametric bias and facial landmarks,” in Proceedings of the IEEE International Conference on Computer Vision Workshops (Venice), 3065–3072.

[B26] Kaulard K. Cunningham D. W. Bülthoff H. H. Wallraven C. (2012). The MPI facial expression database—a validated database of emotional and conversational facial expressions. PLoS ONE 7:e32321. 10.1371/journal.pone.0032321 22438875 PMC3305299

[B27] Kollias D. Tzirakis P. Nicolaou M. A. Papaioannou A. Zhao G. Schuller B. (2019). Deep affect prediction in-the-wild: Aff-wild database and challenge, deep architectures, and beyond. Int. J. Comput. Vision 127, 907–929. 10.1007/s11263-019-01158-4

[B28] Kulkarni K. Corneanu C. Ofodile I. Escalera S. Baro X. Hyniewska S. (2018). Automatic recognition of facial displays of unfelt emotions. IEEE Trans. Affect. Comput. 2018, 1–14. 10.1109/TAFFC.2018.2874996

[B29] la Torre De F. Chu W.-S. Xiong X. Vicente F. Ding X. Cohn J. (2015). “Intraface,” in IEEE International Conference on Automatic Face & Gesture Recognition and Workshops, Vol. 1 (Ljubljana). 10.1109/FG.2015.7163082 PMC4918819 27346987

[B30] Li L. Baltrusaitis T. Sun B. Morency L.-P. (2017). “Combining sequential geometry and texture features for distinguishing genuine and deceptive emotions,” in Proceedings of the IEEE International Conference on Computer Vision Workshops (Venice), 3147–3153. 10.1109/ICCVW.2017.372

[B31] Li S. Deng W. (2019). Blended emotion in-the-wild: Multi-label facial expression recognition using crowdsourced annotations and deep locality feature learning. Int. J. Comput. Vision 127, 884–906. 10.1007/s11263-018-1131-1

[B32] Li S. Deng W. Du J. (2017). “Reliable crowdsourcing and deep locality-preserving learning for expression recognition in the wild,” in Proceedings of the IEEE Conference on Computer Vision and Pattern Recognition (Honolulu), 2852–2861. 10.1109/CVPR.2017.277

[B33] Littlewort G. C. Bartlett M. S. Lee K. (2009). Automatic coding of facial expressions displayed during posed and genuine pain. Image Vision Comput. 27, 1797–1803. 10.1016/j.imavis.2008.12.010

[B34] Liu Z. Wang S. (2012). “Posed and spontaneous expression distinguishment from infrared thermal images,” in Proceedings of the 21st International Conference on Pattern Recognition (ICPR2012) (Tsukuba Science City: IEEE), 1108–1111.

[B35] Lowe D. G. (2004). Distinctive image features from scale-invariant keypoints. Int. J. Comput. Vision 60, 91–110. 10.1023/B:VISI.0000029664.99615.94

[B36] Lucey P. Cohn J. F. Kanade T. Saragih J. Ambadar Z. Matthews I. (2010). “The extended cohn-kanade dataset (CK+): a complete dataset for action unit and emotion-specified expression,” in 2010 IEEE Computer Society Conference on Computer Vision and Pattern Recognition-Workshops (IEEE), 94–101. 10.1109/CVPRW.2010.5543262

[B37] Lucey P. Cohn J. F. Prkachin K. M. Solomon P. E. Matthews I. (2011). Painful data: the UNBC-mcmaster shoulder pain expression archive database. Face Gesture 2011, 57–64. 10.1109/FG.2011.5771462

[B38] Lundqvist D. Flykt A. Öhman A. (1998). The Karolinska Directed Emotional Faces (KDEF). CD ROM from Department of Clinical Neuroscience, Psychology Section, Karolinska Institutet, Stockholm, Sweden.

[B39] Lyons M. J. Kamachi M. Gyoba J. (2020). Coding facial expressions with gabor wavelets (IVC special issue). arXiv 2009.05938.

[B40] Mandal B. Lee D. Ouarti N. (2016). “Distinguishing posed and spontaneous smiles by facial dynamics,” in Asian Conference on Computer Vision (Taipei: Springer), 552–566. 10.1007/978-3-319-54407-6_37

[B41] Mandal B. Ouarti N. (2017). “Spontaneous versus posed smiles—can we tell the difference?” in Proceedings of International Conference on Computer Vision and Image Processing (Honolulu: Springer), 261–271. 10.1007/978-981-10-2107-7_24

[B42] Mavadati M. Sanger P. Mahoor M. H. (2016). “Extended DISFA dataset: investigating posed and spontaneous facial expressions,” in Proceedings of the IEEE Conference on Computer Vision and Pattern Recognition Workshops (Las Vegas), 1–8. 10.1109/CVPRW.2016.182

[B43] Mavadati S. M. Mahoor M. H. Bartlett K. Trinh P. Cohn J. F. (2013). DISFA: a spontaneous facial action intensity database. IEEE Trans. Affect. Comput. 4, 151–160. 10.1109/T-AFFC.2013.4

[B44] McDonald P. W. Prkachin K. M. (1990). The expression and perception of facial emotion in alexithymia: a pilot study. Psychosom. Med. 52, 199–210. 10.1097/00006842-199003000-00007 2330392

[B45] Mehu M. Mortillaro M. Bänziger T. Scherer K. R. (2012). Reliable facial muscle activation enhances recognizability and credibility of emotional expression. Emotion 12:701. 10.1037/a0026717 22642350

[B46] Motley M. T. Camden C. T. (1988). Facial expression of emotion: a comparison of posed expressions versus spontaneous expressions in an interpersonal communication setting. West. J. Commun. 52, 1–22. 10.1080/10570318809389622

[B47] Pantic M. Valstar M. Rademaker R. Maat L. (2005). “Web-based database for facial expression analysis,” in 2005 IEEE International Conference on Multimedia and Expo (IEEE), 5 10.1109/ICME.2005.1521424

[B48] Park S. Lee K. Lim J.-A. Ko H. Kim T. Lee J.-I.. (2020). Differences in facial expressions between spontaneous and posed smiles: Automated method by action units and three-dimensional facial landmarks. Sensors 20:1199. 10.3390/s20041199 32098261 PMC7070510

[B49] Petridis S. Martinez B. Pantic M. (2013). The Mahnob laughter database. Image Vision Comput. 31, 186–202. 10.1016/j.imavis.2012.08.014

[B50] Pfister T. Li X. Zhao G. Pietikäinen M. (2011). “Differentiating spontaneous from posed facial expressions within a generic facial expression recognition framework,” in 2011 IEEE International Conference on Computer Vision Workshops (ICCV Workshops) (Barcelona: IEEE), 868–875. 10.1109/ICCVW.2011.6130343

[B51] Racoviţeanu A. Florea C. Badea M. Vertan C. (2019). “Spontaneous emotion detection by combined learned and fixed descriptors,” in 2019 International Symposium on Signals, Circuits and Systems (ISSCS) (Iasi: IEEE), 1–4.

[B52] Roy S. Roy C. Éthier-Majcher C. Fortin I. Belin P. Gosselin F. (2007). Stoic: A Database of Dynamic and Static Faces Expressing Highly Recognizable Emotions. Montréal, QC: Université De Montréal.

[B53] Saito C. Masai K. Sugimoto M. (2020). “Classification of spontaneous and posed smiles by photo-reflective sensors embedded with smart eyewear,” in Proceedings of the Fourteenth International Conference on Tangible, Embedded, and Embodied Interaction (Sydney), 45–52. 10.1145/3374920.3374936

[B54] Sandbach G. Zafeiriou S. Pantic M. Yin L. (2012). Static and dynamic 3D facial expression recognition: a comprehensive survey. Image Vision Comput. 30, 683–697. 10.1016/j.imavis.2012.06.005

[B55] Saxen F. Werner P. Al-Hamadi A. (2017). “Real vs. fake emotion challenge: Learning to rank authenticity from facial activity descriptors,” in Proceedings of the IEEE International Conference on Computer Vision Workshops, 3073–3078. 10.1109/ICCVW.2017.363

[B56] Saxena A. Khanna A. Gupta D. (2020). Emotion recognition and detection methods: a comprehensive survey. J. Artif. Intell. Syst. 2, 53–79. 10.33969/AIS.2020.21005

[B57] Schmidt K. L. Bhattacharya S. Denlinger R. (2009). Comparison of deliberate and spontaneous facial movement in smiles and eyebrow raises. J. Nonverbal Behav. 33, 35–45. 10.1007/s10919-008-0058-6 20333273 PMC2843933

[B58] Simons G. Ellgring H. Smith Pasqualini M. (2003). Disturbance of spontaneous and posed facial expressions in Parkinson's disease. Cogn. Emot. 17, 759–778. 10.1080/02699930302280

[B59] Smith M. C. Smith M. K. Ellgring H. (1996). Spontaneous and posed facial expression in Parkinson's disease. J. Int. Neuropsychol. Soc. 2, 383–391. 10.1017/S1355617700001454 9375163

[B60] Tavakolian M. Cruces C. G. B. Hadid A. (2019). “Learning to detect genuine versus posed pain from facial expressions using residual generative adversarial networks,” in 2019 14th IEEE International Conference on Automatic Face Gesture Recognition (FG 2019) (Lille: IEEE), 1–8. 10.1109/FG.2019.8756540

[B61] Valstar M. Pantic M. (2010). “Induced disgust, happiness and surprise: an addition to the mmi facial expression database,” in Proceedings of 3rd International Workshop on EMOTION (Satellite of LREC): Corpora for Research on Emotion and Affect (Paris), 65.

[B62] Valstar M. F. Gunes H. Pantic M. (2007). “How to distinguish posed from spontaneous smiles using geometric features,” in Proceedings of the 9th International Conference on Multimodal Interfaces (Nagoya), 38–45. 10.1145/1322192.1322202

[B63] Valstar M. F. Pantic M. Ambadar Z. Cohn J. F. (2006). “Spontaneous vs. posed facial behavior: automatic analysis of brow actions,” in Proceedings of the 8th International Conference on Multimodal Interfaces (Banff), 162–170. 10.1145/1180995.1181031

[B64] Van Der Geld P. Oosterveld P. Bergé S. J. Kuijpers-Jagtman A. M. (2008). Tooth display and lip position during spontaneous and posed smiling in adults. Acta Odontol. Scand. 66, 207–213. 10.1080/00016350802060617 18622829

[B65] Walter S. Gruss S. Ehleiter H. Tan J. Traue H. C. Werner P. (2013). “The biovid heat pain database data for the advancement and systematic validation of an automated pain recognition system,” in 2013 IEEE International Conference on Cybernetics (CYBCO) (Lausanne: IEEE), 128–131. 10.1109/CYBConf.2013.6617456

[B66] Wan J. Escalera S. Anbarjafari G. Jair Escalante H. Baró X. Guyon I. (2017). “Results and analysis of chalearn lap multi-modal isolated and continuous gesture recognition, and real versus fake expressed emotions challenges,” in Proceedings of the IEEE International Conference on Computer Vision Workshops (Venice), 3189–3197. 10.1109/ICCVW.2017.377

[B67] Wang S. Liu Z. Lv S. Lv Y. Wu G. Peng P. (2010). A natural visible and infrared facial expression database for expression recognition and emotion inference. IEEE Trans. Multimed. 12, 682–691. 10.1109/TMM.2010.2060716

[B68] Wang S. Wu C. He M. Wang J. Ji Q. (2015). Posed and spontaneous expression recognition through modeling their spatial patterns. Mach. Vision Appl. 26, 219–231. 10.1007/s00138-015-0657-2

[B69] Wang S. Wu C. Ji Q. (2016). Capturing global spatial patterns for distinguishing posed and spontaneous expressions. Comput. Vision Image Understand. 147, 69–76. 10.1016/j.cviu.2015.08.007

[B70] Wang S. Zheng Z. Yin S. Yang J. Ji Q. (2019). A novel dynamic model capturing spatial and temporal patterns for facial expression analysis. IEEE Trans. Pattern Anal. Mach. Intell. 42, 2082–2095. 10.1109/TPAMI.2019.2911937 30998459

[B71] Wu P. Liu H. Zhang X. (2014). “Spontaneous versus posed smile recognition using discriminative local spatial-temporal descriptors,” in 2014 IEEE International Conference on Acoustics, Speech and Signal Processing (ICASSP) (Florence: IEEE), 1240–1244. 10.1109/ICASSP.2014.6853795

[B72] Xu C. Qin T. Bar Y. Wang G. Liu T.-Y. (2017). “Convolutional neural networks for posed and spontaneous expression recognition,” in 2017 IEEE International Conference on Multimedia and Expo (ICME) (Hong Kong: IEEE), 769–774. 10.1109/ICME.2017.8019373

[B73] Zhang L. Tjondronegoro D. Chandran V. (2011). “Geometry vs. appearance for discriminating between posed and spontaneous emotions,” in International Conference on Neural Information Processing (Shanghai: Springer), 431–440. 10.1007/978-3-642-24965-5_49

[B74] Zhang X. Yin L. Cohn J. F. Canavan S. Reale M. Horowitz A. (2013). “A high-resolution spontaneous 3D dynamic facial expression database,” in 2013 10th IEEE International Conference and Workshops on Automatic Face and Gesture Recognition (FG) (Shanghai), 1–6. 10.1109/FG.2013.6553788

[B75] Zhang X. Yin L. Cohn J. F. Canavan S. Reale M. Horowitz A. (2014). BP4D-spontaneous: a high-resolution spontaneous 3D dynamic facial expression database. Image Vision Comput. 32, 692–706. 10.1016/j.imavis.2014.06.002

[B76] Zhao G. Huang X. Taini M. Li S. Z. PietikäInen M. (2011). Facial expression recognition from near-infrared videos. Image Vision Comput. 29, 607–619. 10.1016/j.imavis.2011.07.002

[B77] Zuckerman M. Hall J. A. DeFrank R. S. Rosenthal R. (1976). Encoding and decoding of spontaneous and posed facial expressions. J. Pers. Soc. Psychol. 34:966 10.1037/0022-3514.34.5.966

